# Effects of supervised aerobic and strength training in overweight and grade I obese pregnant women on maternal and foetal health markers: the GESTAFIT randomized controlled trial

**DOI:** 10.1186/s12884-016-1081-y

**Published:** 2016-09-29

**Authors:** Virginia A. Aparicio, Olga Ocón, Carmen Padilla-Vinuesa, Alberto Soriano-Maldonado, Lidia Romero-Gallardo, Milkana Borges-Cósic, Irene Coll-Risco, Pilar Ruiz-Cabello, Pedro Acosta-Manzano, Fernando Estévez-López, Inmaculada C. Álvarez-Gallardo, Manuel Delgado-Fernández, Jonatan R. Ruiz, Mireille N. Van Poppel, Julio J. Ochoa-Herrera

**Affiliations:** 1 Department of Physiology, Faculty of Pharmacy and Institute of Nutrition and Food Technology, University of Granada, Granada, Spain; 2 Department of Public and Occupational Health, and EMGO+ Institute for Health and Care Research, VU University medical center, Amsterdam, The Netherlands; 3 Department of Obstetrics and Gynecology, University of Granada, Granada, Spain; 4 Obstetrics and Gynecology Service, University Hospital Complex, Granada, Spain; 5 Department of Education, Faculty of Education Sciences, University of Almería, Almería, Spain; 6 Department of Physical Education and Sports, Faculty of Sport Sciences, University of Granada, Granada, Spain; 7 Department of Clinical and Health Psychology, Faculty of Social and Behavioral Sciences, Utrecht University, Utrecht, The Netherlands; 8 Institute of Sport Science, University of Graz, Graz, Austria

**Keywords:** Physical activity, Physical fitness, Nutrition, Glycaemic profile, Oxidative stress, Quality of life, Newborn, Lipid profile, Sleep quality

## Abstract

**Background:**

During pregnancy, a sedentary lifestyle may have negative consequences on maternal and foetal health status. The main objective of this project is to assess the effects of an exercise intervention in overweight and grade I obese pregnant on maternal and foetal health markers.

**Methods/design:**

The present study aims to recruit 60 overweight and grade I obese women interested in participating in an exercise intervention program from the 17th gestational week until delivery. Women will be randomized to either an exercise (three 60-min sessions/week of combined aerobic and strength training and pelvic floor exercises), or usual care (control) group (30 women per group). The primary outcome measures are maternal weight gain, and maternal and neonatal glycaemic profile. Secondary outcome measures are: i) perinatal obstetric records; i) body composition; iii) dietary patterns; iv) physical fitness; v) low-back pain; vi) objectively measured physical activity and sedentary behaviour; vii) haematology and biochemical analyses; viii) oxidative stress; ix) pro- and anti-inflammatory markers; x) bone health biomarkers; xi) sleep quality; xii) mental health, quality of life and positive health.

**Discussion:**

The findings of this project will help to identify strategies for primary prevention and health promotion based on this exercise-based intervention program among overweight and grade I obese pregnant women.

**Trial registration:**

NCT02582567; Date of registration: 20/10/2015

## Background

Assessing the influence of lifestyle during pregnancy on maternal and foetal health is increasingly becoming a matter of interest [1–3]. Among these behaviours, physical activity (PA) may play an important role [4–8]. The sedentary lifestyle adopted by many pregnant women predisposes them to obesity, hypertension or gestational diabetes mellitus (GDM) [5, 6, 8], and the physiological changes occurring during gestation magnify this risk [9]. Importantly, overweight or obesity during pregnancy is associated with a significantly higher risk of pre-term delivery [10], birth-asphyxia-related complications [11], pre-eclampsia, GDM, prolonged labour, caesarean section, wound infection, postpartum haemorrhage, early neonatal death or neonatal intensive care admission [12–15], and infant mortality [16].

Maternal levels of PA may decline during pregnancy likely as a result of the physical changes of pregnancy and due to a combination of social and psychological factors, such as the thinking that resting during pregnancy is the safest behaviour [17, 18]. However, increasing PA levels during pregnancy is effective in the prevention of GDM, hypertension, dysnea, excessive gestational weight gain, and high birth weight, among others [5, 7, 8, 19, 20]. Emerging evidence suggest that exercise training during pregnancy (including moderate-to-high intensity exercise) might provide beneficial effects on both maternal and foetal health without side effects [21, 22]. Indeed, it has been shown that exercise during gestation prevents diastasis recti abdominis [23] and that higher levels of strength or aerobic training are positively associated with hospital stay length, incidence of cesareans and Apgar test [24]. Further, strength training may reduce the need for insulin in overweight pregnant with GDM [25]. Finally, aerobic and strength exercise improve physical fitness and result in additional benefits [26, 27]. Moreover, during pregnancy, not only the DNA of a new life is created, also programmed through epigenetics. In this sense, maternal exercise may have benefits on the newborn, such as higher neurodevelopment [28, 29], better heart functioning, improved heart rate variability [30] and less body fat [28]. However, it is unknown the extent to which supervised exercise programs might improve inflammatory markers, antioxidant activity or bone biomarkers. Moreover, most of exercise programs conducted in pregnant women are performed at light-to-moderate exercise intensity, or are based on solely aerobic or strength training.

An optimal mental health during pregnancy is also a major concern. High levels of depression or anxiety during gestation and post-partum affect maternal quality of life, and in turn, could have a negative influence on the foetal and child health [31, 32]. Exercise may improve the pregnant quality of life and reduce stress [33–36], which might protect the foetus [31]. Also, adequate sleep during gestation improves quality of life [37], prevents stress and depression [38–40] and GDM [41, 42], reduces inflammatory signal [43] and the incidence of preeclampsia, premature birth or caesarea [44–46]. Further, optimal sleep in pregnancy is essential for the foetal development [47] and a better mother-child relationship [40]. However, pregnancy frequently affects sleep quality [48, 49] and it would of clinical relevance to better understand whether an exercise program positively influences the pregnant sleep quality.

Therefore, the main objective of the GESTAtion and FITness (GESTAFIT) project randomized controlled trial (RCT) is to assess the effects of a novel supervised exercise intervention developed in overweight and grade I obese pregnant on maternal and foetal health.

The present methodological article describes the study design, procedures and methods that will be conducted in the “GESTAFIT project”.

## Methods/design

### Study design

**Fig. 1:**
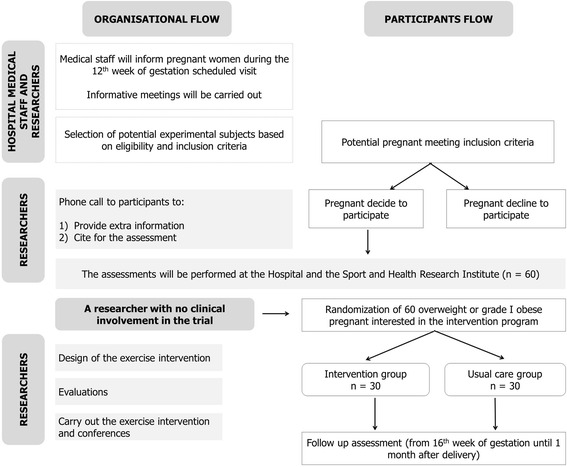
Organizational and participants flow

A supervised RCT (registration number: NCT02582567) based on an exercise program will be conducted in 60 overweight (body mass index (BMI) ≥ 25–29.9), or grade I obese (BMI ≥ 30–34.9) pregnant women (*n* = 30 in the exercise intervention group vs. 30 controls) from Granada (southeaster Spain). The organizational and participants flow is presented in Fig. 1.

The inclusion and exclusion criteria are described in Table 1. Participants will have to provide a written informed consent before taking part in the study, which will be conducted in accordance with the CONSORT (Consolidated Standards of Reporting Trials) statement [50]. Moreover, women will have to answer “No” to all questions from the PARmed-X for pregnancy health checklist (Physical Activity Readiness Medical Examination) by the Canadian Society for Exercise Physiology [51].

**Table 1:** Inclusion and exclusion criteria in the GESTAFIT project

Inclusion criteria	
- Overweight or grade I obese pregnant woman aged 25–40 years old with a normal pregnancy course.	
- Answer “no” to all questions on the PARmed-X for pregnancy*.	
- To be able to walk without assistance.	
- To be able to read and write enough.	
- Informed consent: To be capable and willing to provide consent.	
*In addition, specific inclusion criteria for data analysis are: gestational age at delivery of 37–42 weeks with single foetus, spontaneous vaginal delivery or instrumental vaginal and caesarean without maternofoetal pathology (or other indication that does not involve maternofoetal risk, such as disproportion, failed induction, no foetal progression or non-cephalic presentation), newborn with appropriate weight, Apgar score > 7 in the 1st and 5th minute of life, cord blood pH (normal > 7.20) and normal monitoring results.	
Exclusion criteria	
- Acute or terminal illness.	
- Malnutrition.	
- Inability to conduct tests for assessing physical fitness or exercise during pregnancy.	
- Underweight, normal-weight or grade II-III obesity.	
- Pregnancy risk factors (such as hypertension, type 2 diabetes, etc.).	
- Multiple pregnancies.	
- Chromosopathy or foetal malformations.	
- Uterine growth restriction.	
- Foetal death.	
- Upper or lower extremity fracture in the past 3 months.	
- Presence of neuromuscular disease or drugs affecting neuromuscular function	
- Be registered in other exercise program	
- Perform more than 300 min of at least moderate physical activity per week	
- Unwillingness to either complete the study requirements or to be randomised into control or intervention group.	

PARmed-X for pregnancy: Physical Activity Readiness Medical Examination

This study was approved by the Clinical Research Ethics Committee of Granada, Government of Andalusia, Spain (code: GESFIT-0448-N-15). The study will be conducted following the ethical guidelines of the Declaration of Helsinki, last modified in 2013.

## Recruitment process and measurements procedures

The evaluation protocol scheme is shown in Table 2. The measurements will be carried out in different days. The first assessment day will coincide with the 12th gestational week visit to the gynaecologist at “San Cecilio” University Hospital, Granada, and potential participants will be individually informed about the study objectives, evaluation protocol and procedures. If the woman agrees to participate in the present RCT, the researcher will provide detailed information about each of the phases of the study, and the participant will be asked to read and sign written informed consent. Subsequently (on the same day), weight and height will be assessed. On the second assessment day (16th gestational week) participants will attend the research centre and they will complete the following assessments: nutritional and clinical information, blood pressure, resting heart rate and physical fitness. The initial survey (anamnesis) will be conducted through face-to-face interviews by trained staff in order to gather data on sociodemographic characteristics, reproductive history, history of illness (hypertension, diabetes, obesity, etc.), and diet. Other health information will be collected using a self-administered questionnaire and it will include personal questions regarding smoking and alcohol habits and indicators of socio-demographic and socio-economic status (such as personal and household income, educational level or marital status). This survey will also include questions regarding the employment of nutritional supplements or special diets. Participant will then receive the accelerometer and questionnaires to be completed there or at home, and will be asked to return them eight days later. Additionally, the Hospital will make an appointment with the participants during that week for the biochemical and urine samples collection. The third day of evaluation will consist on accelerometry and sleep quality assessment on the 24th week of gestation. The fourth day of evaluation will take place on the 34th week of gestation. This evaluation will be exactly the same to that performed in the second day of evaluation before the exercise program started (i.e. 16th gestational week), including biochemical assessments.

**Table 2:** Evaluation protocol scheme

	Criteria (12th week)	Baseline (16th week)	Second trimester (24th week)	Third trimester (34th week)	Delivery	1 month post-partum
Informed consent	X					
1st day evaluation						
Sociodemographic data		X				
Clinical history		X		X	X	X
Weight and height	X	X		X		X
Body composition (dual-energy x-ray absorptiometry)						X
Accelerometry		X	X	X		
Physical fitness		X		X		
Nutritional study		X		X		X
Questionnaires to be fill there or at home between the 1st and 2nd evaluation days						
Low back-pain		X		X		X
Quality of life		X		X		X
Sleep quality		X	X	X		X
Physical activity		X	X	X		X
Self-perceived physical fitness		X	X	X		X
Sexual function		X		X		X
Restless legs syndrome		X		X		X
Mental and positive health		X		X		X
2nd day evaluation: Accelerometers collection and review of questionnaires		X		X		
Maternal biochemical analysis		X		X	X	
Umbilical cord blood sampling					X	
Maternal urine sampling		X		X	X	

Blood samples (5 mL) of all pregnant participants will be extracted at week 16th and 34th of gestation, and at delivery. Samples of venous and arterial blood from the umbilical cord (5 + 5 mL) at the time of delivery will be taken. In addition, samples of urine at 16 and 34 weeks gestation and delivery will be collected. All samples will be immediately frozen and conserved at −80 °C to avoid breaking the cold chain before being sent to the laboratory.

## Sample size

The number of participants to be included in the study was calculated on the basis of the change in body weight. We used the difference in weight change observed between the control and intervention group (1.04 Kg) in Ruiz et al. [20] as the expected effect size. A total of 52 women (26 per group) will be needed to detect a mean group difference of 1.04 and a standard deviation of 1.15 Kg in weight change with a power of 90 % and α of 0.05. However, we will exceed this sample size to allow for withdrawals. Assuming a maximum lost-to-follow up of 15 % [20], we will recruit a total of 30 participants per group (*n* = 60).

## Randomization and blinding

After all the baseline evaluations, participants will be randomized to either the exercise intervention or the control group. A computer generated simple randomization sequence will be created before participants will be enrolled, to allocate participants to either group (1:1). The randomization sequence will be prepared by a member of the research team with no clinical involvement in the trial. The allocation will be concealed in a password protected computer file. Whereas the participants will be aware of their group allocation, outcome assessors and data analysts will be blinded to the allocation.

## Exercise intervention

Most of exercise programs developed for pregnant women until now [20, 27, 52] have been designed in compliance with the recommendations of the American College of Obstetrics and Gynaecology (ACOG) in 2002 [53], or the Canadian Society for Exercise Physiology, among others [54]. However, in 2011, Zavorsky & Longo [55] launched a more actual, specific, and evidence-based guideline for exercise programs during pregnancy. Consequently, the exercise program that will be conducted in the present project has been developed accordingly to this guideline [55]. Nonetheless, we have also followed similar studies [56, 57] where aerobic and resistance training, as well as strengthening of the pelvic floor muscles [58], have been developed successfully and in line with these recommendations [57].

The exercise intervention (*n* = 30) will be performed in three groups of 10 participants and will meet the training standards of the American College of Sports Medicine [59] for adults. The groups will train 3 days/week (55–60 minutes per session) from the 17th week of gestation until delivery. The exercise sessions will be designed, carefully supervised, guided and instructed by qualified exercise professionals with experience in working with pregnant women.

The exercise program planning is shown in Table 3. The exercise intervention group will have different phases: a) *Information phase*: which will involve the understanding of the intervention plan for them, the goals we want to work with them, and how we will perform it (one training session); b) *Movement Learning phase*: theoretical and practical sessions with the explanation of movements and explanation of basic movement patterns (two training sessions); and c) *Physical Fitness Training phase*: with training sessions aimed at improving fitness and weight management (from the 18th until the 34th gestational week), and training sessions focused on a correct pelvic mobilization for the delivery (after the 34th gestational week until delivery) in order to try to reduce caesarean section rate, delivery time and number of pushes [23, 60].

**Table 3:** Supervised exercise intervention program

SESION STRUCTURE	CONTENT										
WARM-UP 10 min	Joint mobility and different walk modalities										
CONDITIONING 40 min	Training week	1	2–4	5–6	7–8	9–10	11–12	13–14	15–16	17–18	>19
	Gestational week	17	18–20	21–22	23–24	25–26	27–28	29–30	31–32	33–34	>34
	Intensity (RPE)		12–13	12–13	13–14	13–14	14–15	14–15	15–16	15–16	
	Monday CIRCUIT (muscular and cardiovascular blocks)	Familiarization and acquisition of the basic movement patterns	5 RE × 3	5 RE × 3	5 RE × 3	5 RE × 3	5 RE × 3	5 RE × 3	5 RE × 3	5 RE × 3	Pelvic movements + integration pattern. Real transfer to delivery moment
			1 min REST	1 min REST	1 min REST	1 min REST	1 min REST	1 min REST	1 min REST	1 min REST	
			1 AE 5′	1 AE 5′	1 AE 5′	1 AE 5′	1 AE 5′	1 AE 5′	1 AE 5′	1 AE 5′	
			1 min REST	1 min REST	1 min REST	1 min REST	1 min REST	1 min REST	1 min REST	1 min REST	
			5RE ×3	5RE ×3	5RE ×3	5RE ×3	5RE ×3	5RE ×3	5RE ×3	5RE ×3	
			1AE 5′	1AE 5′	1AE 5′	1AE 5′	1AE 5′	1AE 5′	1AE 5′	1AE 5′	
			1 min REST	1 min REST	1 min REST	1 min REST	1 min REST	1 min REST	1 min REST	1 min REST	
	Wednesday (cardiovascular block)	Familiarization and acquisition of the basic movement patterns	Choreographies and aerobic exercises								Pelvic movements + integration pattern. Real transfer to delivery moment
	Friday CIRCUIT (muscular and cardiovascular blocks)	Familiarization and acquisition of the basic movement patterns	5 RE × 3	5 RE × 3	5 RE × 3	5 RE × 3	5 RE × 3	5 RE × 3	5 RE × 3	5 RE × 3	Pelvic movements + integration pattern. Real transfer to delivery moment
			1 min REST	1 min REST	1 min REST	1 min REST	1 min REST	1 min REST	1 min REST	1 min REST	
			1 AE 5′	1 AE 5′	1 AE 5′	1 AE 5′	1 AE 5′	1 AE 5′	1 AE 5′	1 AE 5′	
			1 min REST	1 min REST	1 min REST	1 min REST	1 min REST	1 min REST	1 min REST	1 min REST	
			5RE ×3	5RE ×3	5RE ×3	5RE ×3	5RE ×3	5RE ×3	5RE ×3	5RE ×3	
			1AE 5′	1AE 5′	1AE 5′	1AE 5′	1AE 5′	1AE 5′	1AE 5′	1AE 5′	
			1 min REST	1 min REST	1 min REST	1 min REST	1 min REST	1 min REST	1 min REST	1 min REST	
COOL-DOWN 10 min	Myofascial release, stretching and relaxation exercises										

*RPE* rating of perceived exertion, *RE* resistance exercise, *AE* aerobic exercise, *REST* resting. The load will be gradually and individualized increased for each participant to reach the intensity designed for each session

The exercise intensity will be prescribed as percentages of heart rate reserve (% HRR) [55] and the ratings of perceived exertion (RPE) using the Borg 6–20 RPE scale [61]. We will also employ the Karvonen formula to estimate the training heart rate (e.g. 65 % HRR for a pregnant with 185 bpm maximum heart rate and 80 bpm basal heart rate would be calculated as: Training Heart Rate = 0.65 * (185–80) + 80). Heart rate will be measured with heart rate monitors (Polar Electro OY, Finland) to control the intensity of the sessions. One third of the participants in the intervention group will wear heart rate monitors in 1/3 of the sessions, both randomly selected. Intensity (expressed as RPE) will be expected to range from 12 to 16.

The intensity will be adapted during the exercise program based on the week of gestation and each woman’s heart rate. The *Physical Fitness Training phase* sessions until the 34th gestational week will consist of mixed work, composed of circuits where both muscular and cardiovascular conditioning will be implemented. This type of exercise training has been already developed by White et al. [57] who demonstrated better results for both the prevention of GDM, preeclampsia and preterm births, and for increased vaginal vs. caesarean deliveries.

Each training session will include 10 min warm-up with walks, mobility and activation exercises. The main part will consist of 40 min exercises organized in circuit. The circuit will alternate muscular and cardiovascular blocks of concurrent training. Each muscle circuit will consist of a hip dominant movement exercise (e.g. deadlift exercises, hinge hip, swing hip), a dominant knee (e.g. squats, lunges) 2 pull movements, 1 push movement (push-ups adapted) and 1 CORE muscles movement. Cardiovascular blocks will take approximately 3 min and will be composed of aerobic exercises as different variants of step-ups, small choreographies, front and side trips. Sessions will finish with a 10 min cool-down period of stretching, breathing, relaxation exercises and myofascial relief [62]. None of the proposed exercises will include Valsalva manoeuvre, supine positions, or high impact exercises that could go in detriment of the successful activation of the lumbo-abdominal belt, promote a decrease in venous return or alterations in blood pressure, among others. The exercise program volume and intensity programmed is also shown in Table 3.

To maximize adherence, several strategies will be implemented including music in all sessions and telephone calls following missed sessions. The researchers will control and register the presence of adverse events during the class and between classes.

The participants randomly assigned to the control group (*n* = 30) will receive general advices about the positive effects of PA during pregnancy for her and the foetus’s health status. The researchers will give three seminar explaining 1) the benefits of exercise for a better pregnancy, prevention and treatment of GDM and excessive weight gain; 2) ergonomic advises, exercises to perform at home (e.g. stretching, resistance training) and strategies to increase their daily PA levels; 3) the benefits of the Mediterranean Diet and nutritional education. Moreover, we will prepare brochures describing the overall benefits of PA on health, and we will dispense general guidelines to increase the level of daily PA and optimum nutrition during pregnancy [63, 64].

## Primary and secondary outcomes

### Primary outcome

The primary outcomes are maternal weight gain and maternal and foetal glycaemic profile. Maternal weight gain will be defined as the weight change from baseline measurement to the last measurement at 34th week of gestation. Maternal insulin sensitivity will be derived from the homeostatic model assessment for insulin resistance (HOMA-IR), which will be calculated using the formula [fasting insulin (μIU/mL) × fasting glucose (mg/dL)]/405 in the 34th week of gestation. Neonatal insulin sensitivity will be assessed through the cord blood ratio glucose/insulin.

### Secondary outcomes

#### Maternal and foetal outcomes

Birth weight; gestational age; type of delivery (natural, instrumental, or caesarean); Apgar scores (at 1 and 5 min); time of dilation, expulsion, and childbirth; GDM; and hypertension, will be obtained from perinatal obstetric records.

#### Blood pressure and resting heart rate

Systolic and diastolic blood pressure, as well as resting heart rate, will be measured after 5 min of rest, on 2 separate occasions (with 2 min between trials), with the person seated (Omron Health Care Europe B.V. Hoolddorp). The lowest value of the two trials will be selected for the analysis.

#### Maternal and foetal anthropometry and body composition

##### Maternal

Height will be measured only at the baseline measurement with a stadiometer (Seca 22, Hamburg). Pre-pregnancy weight will be based on self-report. Height measurement and pre-pregnancy weight will be used to calculate BMI (weight[Kg]/height[m2]) [65].

One month after delivery (postpartum period) lean, fat and bone mass of the whole body will be measured using a dual-energy x-ray absorptiometry (DXA) device (Hologic Discovery QDR, Nasdaq: HOLX). Waist circumference (cm) will be assessed at the middle point between the ribs and the ileac crest, with the participant standing (Harpenden anthropometric tape, Holtain Ltd).

##### Foetus/newborn

A monitoring of the evolution of the foetal anthropometrics will be performed by obstetrical ultrasound measurements following the standards of the International Society of Ultrasound in Obstetrics and Gynaecology guidelines and the methodology by Perin et al. [66]. The baby also will have a physical examination within 24 h after birth. Baby’s weight, length and head circumference will be measured. Newborns will be classified as having low birth weight or macrosomia when birth weight was less than 2500 g and greater than 4000 g, respectively.

#### Dietary patterns

The *Mediterranean Diet Score* [67] in a version adapted to the specific needs during pregnancy for Fe, Ca and folic acid [68] will be used to assess the adherence to the traditional Mediterranean dietary pattern. A moderate alcohol diet, also typical of the Mediterranean Diet, will not be considered for calculating the index in this group of women, who should not drink alcohol due to their pregnancy.

The *frequency of consumption* of foods will be also studied by means of a quantitative questionnaire, validated and designed by Mataix et al. [69].

#### Physical fitness

Participants’ physical fitness status in the 16th and 34th week of pregnancy will be assessed by means of the following tests:

##### Cardiorespiratory fitness

Aerobic capacity will be estimated with a submaximal cycle ergometer heart rate method. A pedaling rate of 50 rpm will kept constant by use of a metronome. Initial workload, 50 W or 75 W, will be based on the women’s reported physical activity levels, and increased by 25 W per minute until a steady state heart rate of 125 · beats or more · per minute was reached, after which the women will cycle for at least 6 min until two consecutive heart rates, one minute apart, differ by 3 or fewer beats · per minute. The VO_2max_ will be calculated according to the Åstrand and Ryhming nomogram [70].

We will additionally perform the *6*-*min walk* test, which measures the maximum distance (in meters) each participant can walk in 6 min along a 45.7 m rectangular course [71].

##### Upper-body muscle strength

The *handgrip strength* will be measured using a digital dynamometer (TKK 5101 Grip-D; Takey, Tokyo, Japan) as described elsewhere [72]. The participants will perform (alternately with both hands) these tests twice. The best value of 2 trials for each hand will be chosen and the average of both hands will be used in the analyses.

##### Upper-body flexibility

The *back*-*scratch* test, a measure of overall shoulder range of motion, involves measuring the distance between (or overlap of) the middle fingers behind the back with a ruler [71]. The best score of two attempts for each arm in centimetres will be recorded and the average of both arms will be used in the analyses.

##### Self-perceived physical fitness

The *International Fitness Scale* [73] is composed of five Likert-scale questions asking about the perceived participants’ overall fitness, cardio-respiratory fitness, muscular fitness, speed-agility and flexibility in comparison with their friends’ physical fitness (“very poor”, “poor”, “average”, “good” and “very good”).

#### Low-back pain

Pregnant women usually report low-back pain, especially in the third gestational trimester. Low-back pain will be assessed with the Spanish version of the *Oswestry Disability Index score* [74]. We will additionally employ the Pain Visual Analogue Scale to assess low back pain intensity [75].

#### Physical activity and sedentary behaviour

*Accelerometry* will be used to objectively assess PA and sedentary time. Women will be asked to wear a tri-axial accelerometer (ActiSleep+, Pensacola, Florida, United States) for 9 consecutive days, starting the same day they receive the monitor (e.g. participants who receive the accelerometer on Monday, will carry the device until Tuesday of the next week). The first and last day will be excluded from the analyses, accounting for a total of 7 days of registering. Participants will be instructed to wear the accelerometer during the whole day (24 h) on their wrist attached by an elastic belt. To prevent any damage to the devices, these will be taken off during water-based activities such as bathing or swimming. Time engaged in light, moderate, and moderate-vigorous intensity PA and sedentary time will be calculated. This accelerometer has been previously used in pregnancy with similar methodology as described in the present protocol [19].

#### Haematology and biochemical analysis

Venous blood samples will be extracted in standardized fasting conditions at 8–9 a.m. in the hospital and collected in EDTA vacuum tubes, and tubes for serum. Some samples will be centrifuged at 1750 rpm for 10 min at 4 °C in a refrigerated centrifuge (GS-6R Beckman, Fullerton, CA, USA) to separate plasma from formed elements. Subsequently, erythrocytes will be centrifuged in a hypertonic solution to remove membranes and cytosol.

##### Haematology

Erythrocyte count, haematocrit, haemoglobin, platelets, leukocytes and erythrocyte mean corpuscular volume will be quantified by a Coulter counter.

##### Biochemical parameters

Plasma total, high-density lipoprotein and low-density lipoprotein cholesterol, triglycerides, phospholipids, creatine kinase, aspartate aminotransferase, alanine aminotransferase, gamma-glutamyl transferase, lactate dehydrogenase, alkaline phosphatase, glucose, urea, insulin, albumin, high sensitivity C-reactive protein, glycosylated haemoglobin, creatinine, total bilirubin, myoglobin, lactate, troponin I, peroxisome proliferator-activated receptor-γ coactivator (PGC)-1α, nitric oxide and myeloperoxidase will be assessed with standard methods using an autoanalyzer (Hitachi-Roche p800, F. Hoffmann-La Roche Ltd. Switzerland) or by using commercial kits (spectrophotometry and ELISA).

#### Oxidative stress

##### a) Antioxidant defence

- Plasma measurements: liposoluble antioxidants (vitamin E, retinol, carotene, coenzyme Q10 and coenzyme Q9) through mass spectrometry and the total plasma antioxidant capacity by using commercial kit (spectrophotometry).
- Erythrocyte measurements: liposoluble antioxidants in membrane (vitamin E and coenzyme Q10) through mass spectrometry and antioxidant enzymes activity (catalase, glutathione peroxidase and superoxide dismutase) by spectrophotometry.

##### b) Oxidative damage

- Oxidative damage to proteins: measurement of carbonyl proteins by using commercial kit (spectrophotometry).
- Oxidative damage to lipids: measurement of 4-hydroxynonenal and isoprostanes in urine and plasma and hydroperoxides in plasma and erythrocyte membrane by using commercial kits (spectrophotometry and ELISA).
- Oxidative damage to DNA: measurement of 8-hydroxyguanosine in urine and plasma by commercial kits (ELISA).

#### Pro- and anti-inflammatory signal

Some maternal and umbilical cord plasma pro-inflammatory and anti-inflammatory cytokines (IL-1β, IL-2, IL-6, IL-8, IL-10, IFN-γ and TNF-α, IL-1ra and TNF Srii α), some adipokines (adiponectin, adipsin, resistin, PAI-active, insulin and leptin) and myokines (irisin) will be measured by the employment of Luminex xMAP technology.

#### Bone biomarkers

Various relevant biomarkers related to bone metabolism (ACTH, DKK-1, FGF-23, Osteocalcin, OPN-Osteopontin, Osteoprotegerin, PTH and SOST) will be measured with Luminex xMAP technology.

#### Sleep quality

The *Pittsburgh Sleep Quality Index* [76] will be used to assess sleep quality and disturbances over a l-month time interval. Nineteen individual items generate seven “component” scores: subjective sleep quality, sleep latency, sleep duration, habitual sleep efficiency, sleep disturbances, use of sleeping medication, and daytime dysfunction. Each component yields a score ranging from 0 to 3, with 3 indicating the greatest dysfunction. The seven component scores are summed to provide a global sleep quality score (range 0 to 21) with higher scores indicating poor sleep quality.

Nevertheless, it has been shown that reported sleep by pregnant women varies in relation to that objectively estimated with accelerometry [77]. Consequently, behaviours related to sleep, such as sleep onset, sleep latency, total sleep time, number and duration of awakenings and sleep efficiency will be objectively calculated by using a triaxial accelerometer (ActiSleep+, Pensacola, Florida, United States). The data analysis of such ActiSleep accelerometry data will be carried out through its specific software (Actilife).

#### Quality of life, mental health and positive health

We will use the *Short*-*Form Health Survey 36* [78], for assessing health-related quality of life. It contains 36 items grouped into 8 dimensions: physical functioning, physical role, body pain, general health, vitality, social functioning, emotional role, and mental health. The scores range from 0 to 100 in every dimension, where higher scores indicate better health.

The pregnant antenatal depression will be assessed with the *Center for Epidemiological Studies*-*Depression Scale* questionnaire [79], which is validated and widely employed in pregnancy. Postnatal depression levels will be assessed with the Spanish version of the *Edinburgh Postnatal Depression Scale* [80], which is a 10-items self-report scale designed as a specific instrument to detect postnatal depression.

Anxiety levels will be assessed with the *State Trait Anxiety Index* [81]. This questionnaire consists of two scales that assess anxiety state and trait anxiety. The total score ranges from 20 to 80, with higher values indicating higher levels of anxiety.

The “Restless Legs Syndrome” largely determines the quality of life for many pregnant and is related to their levels of depression and sleep quality [82]. The Spanish version of the *Detention of Restless Legs Syndrome* questionnaire [83] will be employed to assess the presence and severity of this syndrome.

Sexual function is also an important aspect of the quality of life of a person. The Spanish version of the *6*-*item Female Sexual Function Index* [84] was used to assess sex function. This instrument is composed of six questions: desire, arousal, lubrication, orgasm, satisfaction and pain. Each question can be scored from 0 to 5 and then summed up to provide a total score.

Finally, “positive health” will be evaluated through the following questionnaires:

1. The *Trait Meta-Mood Scale* [85] is comprised of 3 subscales to assess participant’s beliefs about attending to and value their feelings (emotional attention), feel clear rather than confused about their feelings (emotional clarity) and how well participants regulate their moods and repair negative emotional experiences (emotional repair). Each subscale comprises 8 items. Participants rate their responses using a 5-point Likert type scale, with 1 = “strongly disagree” to 5 = “strongly agree”. The subscales score range from 8 to 40 and higher scores reflect greater attention, clarity, and repair.
2. The *Positive and Negative Affect Schedule* [86] is a 20-item questionnaire designed to measure positive and negative affect. The questionnaire includes 10 positive and 10 negative emotional states that should be answered on a 5-point Likert scale, from 1 = “very slightly or not at all” to 5 = “extremely”. The score ranges from 10 to 50 for both subscales (positive affect and negative affect), and higher scores reflect greater affective well-being.
3. The *10*-*item Connor-Davidson Resilience Scale* [87, 88] assesses resilience to stress, which is a construct refers to a dynamic process of positive adaptation to adverse changes in life circumstances. Each item ranges from 0 = “not true at all” to 4 = “true nearly all the time”. The total score range from 0 to 40, and higher scores indicate greater resilience.

## Statistical analysis

Since this study aims at determining potential biological effects of exercise, the statistical analysis will be performed on a per-protocol basis, thus, including in the analyses only participants who attended at least 80 % the exercise session and completed both baseline and follow-up evaluations. The treatment effects will be reported as between-group changes on the primary and secondary outcomes and will be assessed with generalized linear models after adjusting for baseline values. All the analyses will be repeated additionally adjusting for age and any other confounder potentially not well balanced at baseline, and the results from both models will be reported. The effect size (95 % confidence interval) and statistical significance will be reported for each study outcome. The statistical significance will be set at α = 0.05. Sensitivity analyses will be conducted using intention-to-treat analyses and baseline observation carried forward imputation.

## Discussion

This paper describes the protocol performed by a multidisciplinary team of experts in PA and exercise, nutrition, gynaecology and physiology that aims to determine the effects of a supervised exercise intervention developed in overweight and grade I obese pregnant on the mother and newborn health, and the influence of lifestyle during pregnancy on relevant, but no explored yet, maternal and foetal outcomes.

The intrauterine environment seems to be involved in programming and foetal offspring exposed to maternal stress, inappropriate diet, physical inactivity, obesity or hyperglycaemia, among others, may be prone to future metabolic alterations and chronic diseases [26, 89–91]. Among these modifiable behaviours, PA is especially relevant during gestation [4–6]. Increasing PA levels or exercising during pregnancy is an efficient tool to tackle cardiometabolic diseases such as GDM, and excessive maternal and neonatal weight gains [5, 19, 92]. However, modern society sometimes treats pregnancy as a disease. For example, it qualifies as vulnerable women and are excluded from most studies [93]. Furthermore, is noticeably that most of the interventions focus on improving pregnant health involve mere advices or counselling given face to face, or by phone (or both). Just few trials have investigated well-controlled individually tailored exercise interventions. Consequently, further trials, with objective outcome measures, are needed. Moreover, the influence of physical fitness on important maternal health markers during gestation, such as quality of life, positive health, inflammatory profile, bone health, or oxidative stress is unknown.

Exercise during pregnancy is safe [21, 22] and side-effects are minimal [53, 54] if pregnant adequate the intensity of such exercise to warrant foetal wellbeing [94]. However, the exercise intensity suggested by the ACOG in 2002 [53] and by Davenport et al. [95] will not be adopted in the present RCT. This is firstly, because the minimum work intensity is stated at 101 bpm for all women (which constitute a low heart rate), and secondly, because they make an absolute estimation (independently of the woman’s age, basal heart rate, or physical fitness status). Thereby, in agreement with Zavorsky & Longo [55], we advocate for modulating the intensity based on the % HRR, and the RPE.

Not less important for an optimal pregnancy is a healthy psychological status [31, 32, 96]. In that sense, exercise represent an excellent opportunity to improve quality of life [33] and reduce cortisol levels, which may in turn have a positively influence on the foetus and child [31, 32]. Besides, it is unknown whether exercise could positively influence the characteristic worse sleep quality during gestation [48, 49], which can alter the pregnant metabolic status [41–43] and gestational outcomes [44–46] and negatively affect the pregnant quality of life [37–40] and the foetal development [47].

There is a clear and compelling rationale for increased pregnancy research in order to address the therapeutic needs of pregnant women [93]. Additionally, there is accumulating evidence that pregnancy provides a unique window into understanding fundamental mechanisms underlying observed links between a pregnant woman’s health and her later health and the health of her children [93]. The information obtained from this RCT will therefore be of clinical and public health interest and will suggest future research. Further, the exercise intervention designed is novel and non-expensive, and can be easily transferred to other similar contexts. Consequently, the findings of the GESTAFIT Project will help the Health Systems to identify strategies for primary prevention and health promotion among overweight and obese pregnant.

## Abbreviations

GDM: Gestational diabetes mellitus
HRR: Heart rate reserve
PA: Physical activity
RCT: Randomised controlled trial
RPE: Rate of perceived exertion
SD: Standard deviation
VO_2max_: Maximal oxygen uptake
